# The COVID-19 Pandemic: Does Our Early Life Environment, Life Trajectory and Socioeconomic Status Determine Disease Susceptibility and Severity?

**DOI:** 10.3390/ijms21145094

**Published:** 2020-07-19

**Authors:** Cyrielle Holuka, Myriam P. Merz, Sara B. Fernandes, Eleftheria G. Charalambous, Snehaa V. Seal, Nathalie Grova, Jonathan D. Turner

**Affiliations:** 1 Immune Endocrine Epigenetics Research Group, Department of Infection and Immunity, Luxembourg Institute of Health, L-4345 Esch-sur-Alzette, Luxembourg; cyrielle.holuka@lih.lu (C.H.); myriam.merz@lih.lu (M.P.M.); SaraBeatriz.Fernandes@lih.lu (S.B.F.); eleftheria.charalambous@lih.lu (E.G.C.); snehaa.seal@lih.lu (S.V.S.); nathalie.grova@lih.lu (N.G.); 2 Calbinotox, Faculty of Science and Technology, Lorraine University, 54506 Nancy, France

**Keywords:** COVID-19, SARS-CoV-2, socioeconomic status, early life adversity, psychosocial stress, immunosenescence, immune exhaustion, health inequalities

## Abstract

A poor socioeconomic environment and social adversity are fundamental determinants of human life span, well-being and health. Previous influenza pandemics showed that socioeconomic factors may determine both disease detection rates and overall outcomes, and preliminary data from the ongoing coronavirus disease (COVID-19) pandemic suggests that this is still true. Over the past years it has become clear that early-life adversity (ELA) plays a critical role biasing the immune system towards a pro-inflammatory and senescent phenotype many years later. Cytotoxic T-lymphocytes (CTL) appear to be particularly sensitive to the early life social environment. As we understand more about the immune response to SARS-CoV-2 it appears that a functional CTL (CD8+) response is required to clear the infection and COVID-19 severity is increased as the CD8+ response becomes somehow diminished or exhausted. This raises the hypothesis that the ELA-induced pro-inflammatory and senescent phenotype may play a role in determining the clinical course of COVID-19, and the convergence of ELA-induced senescence and COVID-19 induced exhaustion represents the worst-case scenario with the least effective T-cell response. If the correct data is collected, it may be possible to separate the early life elements that have made people particularly vulnerable to COVID-19 many years later. This will, naturally, then help us identify those that are most at risk from developing the severest forms of COVID-19. In order to do this, we need to recognize socioeconomic and early-life factors as genuine medically and clinically relevant data that urgently need to be collected. Finally, many biological samples have been collected in the ongoing studies. The mechanisms linking the early life environment with a defined later-life phenotype are starting to be elucidated, and perhaps hold the key to understanding inequalities and differences in the severity of COVID-19.

## 1. Introduction

The ongoing outbreak of coronavirus disease (COVID-19) was first reported in December 2019 in Wuhan, China. COVID-19 is caused by a betacoronavirus, severe acute respiratory syndrome coronavirus 2 (SARS-CoV-2), that affects the respiratory system [1]. Despite draconian sanitary measures being applied worldwide, COVID-19 was declared a pandemic on 11 March 2020 by The World Health of Organization (WHO) [2]. By May 13th the outbreak had infected over 4 million people and caused almost 300,000 deaths worldwide (World Health of Organization, 2020).

There is a long-established epidemiological observation that social adversity associates with reduced host resistance to infection and disease [3] which goes back as far as 1976 [4]. More recently, it was recognized that the effect on adult immune function and disease risk was much stronger when the exposure to adversity occurred during early life [5,6]. Humans are not fully developed at birth. Nervous and immune systems are gradually developed and educated up to the age of two. In fact, human life commences and develops for the first 1000 days starting from fetal conception. Any pre-natal complications and post-natal adversity faced defines the lifelong health trajectory [7]. As the COVID-19 pandemic has progressed, it has become clear there are many inequalities in susceptibility and severity of the disease. The recent flurry of pre-print clinical data from many countries worldwide including China, UK, US, are strongly concordant; the lower the current socioeconomic status (SES), the greater the risk [8], however, the role of the early life period and the resultant life-course has so far not been investigated. To understand the mechanisms underlying these differences, we need to dissect the exposome and environmental factors (i.e., pollutants, stress situation, etc.) that patients may be, or have previously been exposed to.

There is a well-established literature on the role of the overall trajectory from early life through to adulthood and the risk of non-communicable diseases such as cardiovascular disease, diabetes, obesity and depression [9], however there is no data on how it affects COVID-19. Although current SES has been associated with the risk, progression and even survival of non-communicable diseases [10], it is now becoming clear that during an individual’s life there are periods of increased susceptibility, and the overall trajectory of SES may be more important. This has led to the “Barker theory”, or the Developmental Origins of Health and Disease (DOHaD) [11]. In addition, environmental influences which act during early development/life may determine our susceptibility to the disease many years later [11,12,13].

Over time, the Barker theory has been refined. Currently, this is thought of as a “three hit model”. The three “hits” are generally accepted as: (1) genetic predisposition, (2) early life environment and, (3) later life environment [14,15]. As high-quality mechanistic studies have addressed the link between the early-life period and adult disease, it is becoming clear that the immune system, particularly through chronic low-grade inflammation and accelerated immuno-senescence is, mechanistically, in the heat of the action. In addition, we know that stressful experiences during early life induce adaptive responses that are often mediated by the immune system [16].

In this manuscript, we examine the data linking early life adversity to life-long disturbances in the immune system that may play a role in determining its ability to fight SARS-CoV-2 infection, potentially determining the severity of COVID-19 disease and expanding DOHaD to cover infectious diseases later in life.

Furthermore, we review known factors of ELA and their potential influence on the adult immune system and contemplate what kind of data should be collected to understand how SES and ELA influence disease susceptibility and severity of COVID-19 and other diseases. We hope this work will contribute in protecting and treating people at risk of developing severe COVID-19 symptom.

## 2. The Role of Current SES in COVID-19 Morbidity and Mortality

Socioeconomic status (SES) or gradient is a combination of education, incomes, occupation and reveal inequities to privileges or resources between individuals [10]. Indeed, socioeconomic factors (i.e., race/ethnicity) are considered as fundamental determinants in human life span, well-being and health [10]. Data from influenza pandemics of 1918 and 2009 showed that socioeconomic factors may determine both disease detection rates and overall outcomes [17,18,19]. In the early phase of the COVID-19 pandemic many studies focused on basic criteria (i.e., age, sex, and gender) to investigate coronavirus spread, transmission routes and potential high-risk populations. Socioeconomic data were, unfortunately, missing as they are not considered as data of clinical interest [16]. However, socioeconomic data regroup many relevant factors as daily situations (i.e., stressful job, pollution, etc.) that directly interact with human health [16]. Evidence is now starting to emerge that COVID-19 mortality is increased in ethnic minority populations. US data indicates that, for example, in Chicago approximately 70% of the deaths were from ethnic minorities [20]. Detailed data from New York showed that the number of COVID-19 cases associated with the percentage of dependents in the local population, the male:female ratio, and low-income neighborhoods [21]. United States-wide data gave a similar result, with proportion of residents >65 years old, ethnic minorities, male:females ratio, and the overall population density associating with increased frequency of COVID-19 [22]. The United Kingdom followed a similar profile. Although the recent UK data only looked at mortality, there was a stronger link between COVID-19 mortality and SES than ethnic background. A 1% increase in the lower socioeconomic class increased COVID-19 mortality by 2% (95% Confidence interval of 1% to 4%) while a 1% increase in ethnic minority increase mortality by only 1% (95% confidence interval 1% to 2%) [8]. Although these are preliminary (pre-print) data, they agree with Shi et al., who reported that the most severe cases were mostly agricultural laborers [23]. The link between the incidence of COVID-19 and lower income neighborhoods and lower SES is most likely due to the overall economic conditions such as poverty, performing essential public tasks, poor quality and over-populated housing as well as an obligation to use public transport [8] as well as higher rates of known comorbidities including type 1 and 2 diabetes, as well as cardiovascular disease and hypertension [24]. Overall, despite the scarcity of the data, we interpret what is available as a suggestion that current SES and neighborhood influence the morbidity of SARS-CoV-2 infection and COVID disease rather than the mortality rate.

## 3. The Role of Early Life in Determining Lifelong Health Trajectories

**Figure 1:**
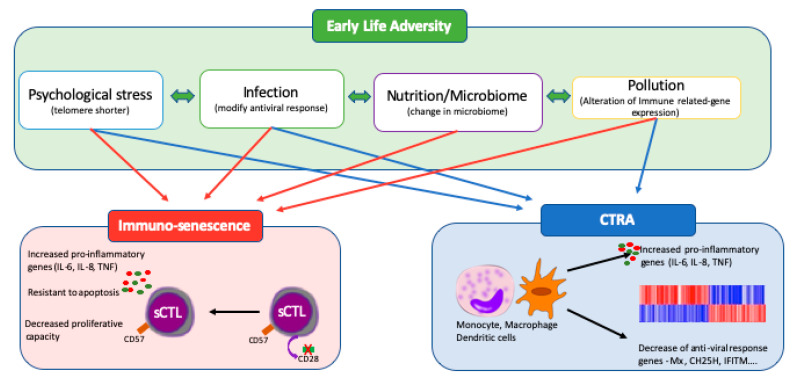
Immune adaptation mediated by early life adversity. Early-life adversity (ELA) is broken down into its four key components: psychosocial stress, infectious stress, nutrition and the microbiome; and pollutant exposure. They are linked to increases in the numbers of senescent cytotoxic lymphocyte (sCTL) which, upon stimulation are resistant to apoptosis and release large quantities of expression of pro-inflammatory. Certain elements have also been shown to alter the underlying transcriptional identity of leucocytes such as macrophages, dendritic cells or T lymphocytes. This phenomenon is called “the conserved transcriptional response to adversity” (CTRA).

When considering the early-life environment, many measures such as SES are broad and encompass many concurrent elements. We have previously found it useful to separate these into four principal sub-categories [15] (Figure 1). Although determining the contribution of each of the four elements (psychosocial stress, infection, nutrition and microbiome, and pollutant exposure) is difficult, there are data on well-defined exposure conditions that fit into these sub-categories as well as insidious, general measures like SES.

### 3.1. Early Life Psychosocial Stress

There is now a growing literature on the effects of early-life psychosocial adversity on the immune system. We have previously reported the immunophenotype of young adults that had experienced ELA as institutionalization after separation from their parents and subsequently adopted in early childhood compared to those reared by their biological parents (EpiPath cohort) [25]. In this cohort, we surveyed the innate, humoral, and adaptive immune system. We observed an increase in activated and senescent pro-inflammatory T cells, particularly those, expressing HLA-DR/CD25 and CD5. Senescence is a natural aging process affecting all cells including immune cells. These begin to deteriorate and this leads to weakened immune responses [26]. Furthermore, there was a trend toward an increase in the number of circulating Th17 cells [27,28]. ELA clearly accelerated T-lymphocyte maturation and senescence, although did not affect B cells. T- lymphocytes were accelerated through their maturation cycle from naïve to effector memory and aggregating in the terminally differentiated effector memory cells re-expressing CD45RA (TEMRA) cell phase [27,28]. This skewing of the immune system, in particular the cytotoxic CD8+ T-cells was confirmed in an independent cohort, of teenagers approximately 15 years after a similar form of ELA [29].

Telomere length decreases with chronological and biological age, after cell division, and is a hallmark of cellular senescence. Exposure to stressful events during childhood showed that the telomere length is shorter in these individuals when compared to the control group [30,31,32,33], confirming that ELA negatively contributes to an imbalanced immune system [34]. Furthermore, Cohen et al. showed that low childhood SES significantly decreased the telomere length later in life of a CD8+CD28- T cell population, which play major role in the response to viral infections [35].

Studies with rodents produced the predominant hypothesis that the mechanism by which ELA impacts the function of CD8+ cells and, consequently, viral responses, may be through the HPA axis. ELA negatively impacts the HPA axis, which programs its effects and responses later in life. This normally results in a decreased release of corticosterone or cortisol after exposure to stress which consequently has a great impact on the peripheral immune system, leading to compromised viral responses [36,37,38,39]. However, results from mechanistic studies in our EpiPath cohort have excluded this. We were able to show that despite an altered HPA axis [25], glucocorticoid signaling and the peripheral HPA-axis stress system were not epigenetically programed [40], implying that the immune system was directly impacted.

### 3.2. Early Life-Infections

It is well known that an early life exposure to infection and inflammation can have devastating effects. One example would be that neonates suffering from bacterial or viral sepsis are about threefold more likely to die within the first 120 days [41]. There is also evidence showing that sepsis in new-borns was associated with poor long-term neurodevelopment [42]. The immediate risk of infection to the organism, especially for those more vulnerable, seems obvious. The long-term consequences of an infection prove far more difficult to grasp.

Bilbo and Schwarz reviewed available data on the connection between perinatal infection and long-term effects on stress reactivity and cytokine production [43] showing that early life infection leads to a cytokine storm (the most prominent being interleukin 1β [IL-1β], IL-6 and tumor necrosis factor α [TNFα] which can pass the blood-brain-barrier and cause long term memory impairment in the hippocampus. Similarly, we found a blunted response to stress and a higher number of exhausted T-lymphocytes in our EpiPath adoptee cohort, which had a higher incidence of cytomegalovirus (CMV) infection and an overall higher risk of childhood infections due to the institutionalization [25,27]. A very recent study in zebrafish shows that expression of several pro-inflammatory genes is increased in adult fish after early life bacterial infection [44]. This study also showed that the age of the first infection is a crucial factor for the adult immune response. Other studies have specifically linked early-life respiratory viral infection with a higher likelihood to develop diseases like childhood asthma or allergies [45,46,47] or the chance to develop type 1 diabetes [48]. These chronic conditions are known risk factors for a more severe outcome of COVID-19 disease.

Currently, the molecular mechanisms in which an early life infection distorts the immune system are only partially understood. In in-vitro experiments, Fonseca et al. demonstrated that early-life exposure to bacteria in combination with respiratory syncytial virus (RSV) later in life can lead to epigenetic modifications impacting bone marrow progenitor cells and therefore causing long-term re-shaping of inflammatory mediators and metabolic profiles [49]. Subsequently, all daughter cells of these progenitors would be ill-equipped to handle subsequent infections [47].

Certainly, early life infections present a specific type of early life adversity. It is indubitably linked to the overall health of the individual (immune system) and the social environment, given that host-to-host transmission of pathogens are by far the most prevalent form of infection. In the previous section, we showed the impact of psychosocial stress on the immune system. However, the overlap does not end there: sickness, in humans and animals, also changes their social behavior. Well known behavioral changes include a decrease in activity and expanded sleeping periods [50]. Therefore, social behavior and infection should not be treated as two distinct adversities, but as two sides of the same coin.

*Early life nutrition and the microbiome*: Over the last decade it has become clear that once the microbiome is established it is shaped by the exposome and the ~9 million microbial genes it encodes and play a crucial role in determining host development and health [14,51,52,53]. Modulating the host most probably protects the natural enteric symbiotic microbial community, and disturbing the established microbiome, producing a dysbiosis, results in disease and may even be fatal [54,55]. The microbiome established is dependent on the route of birth, and is then modulated by nutritional intake, living conditions, the polluted environment and the presence of pets [56,57]. As SARS-CoV-2 appears to persist in the GI tracts and can be detected in human feces [58,59], it will interact, affect, and be affected by the microbiome. Indeed, diarrhea is now recognized by the Centers for Disease Control and Prevention (CDC) as a COVID-19 symptom and it is a clear sign of microbial dysbiosis [60]. The interaction and effects of SARS-CoV-2 will almost certainly depend on both the microbiome that has been established and how the host has adapted to its microbiome.

The LPS content and immunostimulatory potential of the initial early-life microbiome depends on the birth route [51]. The microbiome is established during a sensitive period in which the new-born immune system is primed [61], and may explain why babies born by caesarean section have a significantly increased risk of allergy or asthma later in life [62]. Exposure of new-borns to a more diverse microbiota soon after birth altered both the disease susceptibility and maturation of specific immune cell subsets, whereas if the first encounter occurred later, immune dysfunction was not corrected [63,64]. Regulatory T cells (T_reg_) play a significant role in the host adaptation to the microbiome, recognize host-specific commensal bacteria derived antigens [65], and result in long-term tolerance to the enteric microbiome [66]. It would appear that adverse microbiota is essential for the immune system to fully mature [67].

Peri-natal viral infections, such as CMV have been extensively studied and linked to lifelong changes in the microbiome [68] and common viruses such as influenza are known to affect the development of the immune system when acquired at birth and during infancy [69]. The angiotensin-converting enzyme 2 (ACE2) receptor may play a role in determining microbiome-immune-interactions. In the GI tract ACE2 is expressed in enterocytes and is important for maintaining both antimicrobial peptide expression, and the overall health of the microbiome [70,71]. Mice lacking Ace2 develop gut absorption related diseases [70,72]. As Sars-Cov-2 uses ACE2 receptor to enter cells [73,74] it would be logical to assume that there is a link between the virus and the microbiome that was established in early life, immune cells resident in the GI tract and the overall outcome of COVID-19.

*Early life-pollution exposure*: There is emerging evidence that environmental exposure to pollutants during sensitive developmental periods like early life could be a strong factor of susceptibility, predisposing the individual to birth outcomes and disease onset in later life [15]. Prenatal exposure to airborne pollutants could affect fetal reprograming by epigenetic modifications (e.g., DNA methylation) and may therefore explain the potential link between air pollutant exposure and adverse pregnancy outcomes. Epidemiological studies have pointed out causal association between fine particulate matter (2.5 μm; PM2.5) and neurodevelopmental (ADHD, autism)/neurodegenerative (Parkinsons, Alzheimers) [15], metabolic, cardiovascular [75] and lung pathologies [76]. Air pollutants were therefore proved to affect key cellular/molecular targets during the perinatal period, which are susceptible to alter immune responses link to abnormal respiratory functions and lung diseases later in life [77]. For instance the EDEN birth cohort study, focusing on determining peri-natal factors that influence childhood health and social development, pointed out that a pre-natal exposure to PM10 (particles with diameter less than 10 µm) was linked to an increased in CD8+ T cell and a decreased in regulatory T cells in infants at birth, leading to a potential increase in the susceptibility of viral infection responses as well as atopy development in children [78]. The impact of traffic pollutants and tobacco smoke on regulation of numerous Immune related-genes, such as cytokines (e.g., IL-4, IL-6, and IFNg), TLR2, nitric oxide synthases (NOSs), and several factors of transcription (e.g., Runx3 and Foxp3), has also been demonstrated [77]. It is now well established that modifications in DNA methylation patterns due to PM 2.5 exposure are frequently associated with the development of lung pathologies [79]. However, it remains difficult to assess whether exposure during early life has a stronger impact on development of diseases than that of the adulthood, or whether substantial morbidity is the result of accumulated exposure [76].

In the context of COVID-19, Zhu et al. demonstrated significant associations between air pollution and COVID-19 infection. High concentration levels of PM_2.5_, PM_10_, CO, NO_2_ and O_3_ were therefore positively linked to a risk of COVID-19 infection, whereas high concentration levels of SO_2_ were negatively linked to the number of daily COVID-19 confirmed cases [80]. These results are supported by those obtained in February 2020 by Martelletti et al., who showed that in the industrialized regions of Northern Italy, those most affected by COVID-19, the concentration levels of PM10 and PM2.5 were above the legislative standard limit of 50 μg per day [81]. The adsorption of SARS-CoV-2 RNA on airborne PM (PM2.5 and PM10) was established in these regions by Setti et al. who suggested that, “in conditions of atmospheric stability and high concentration of PM, SARS-CoV-2 could create clusters with outdoor PM, and, by reducing their diffusion coefficient, enhance the persistence of the virus in the atmosphere.” [82]. In a cross-sectional observational study conducted in the United States, Wu et al. showed, by taking into account 20 potential confounding factors in their main analysis, that a slight increase in PM2.5 (+1 μg/m^3^) was linked to an 8% increase in the rate of COVID-19 death [83]. Although all this data results from preliminary investigations, it tends to suggest a positive relationship between ambient air pollution exposure and COVID-19 mortality rate. Confirming the direct impact of airborne pollutants on the COVID-19 severity could prove an asset in terms of public health and prevention strategy in places with poor air quality.

We have previously highlighted the role of early-life pollution exposure and a potential “second hit” in the “three-hit” model producing a quiescent phenotype, likely encoded in the epigenome, which might become vulnerable in later life to a “third environmental hit” such as COVID-19 [15]. Given the long-term effects on health of early-life pollutant exposure and the linkage with the development and progression of pulmonary pathologies in later-life, it is reasonable to assume that early-life pollutant exposure will affects the course of COVID-19.

## 4. Early Life Origins of COVID Co-Morbidities

If the early life environment plays a role in determining the outcome of COVID-19, examining its role in the key comorbidities is essential. The three key comorbidities determining COVID-19 severity are cardiovascular disease, hypertension and diabetes. The seminal work of David Barker clearly identified the role of the in-utero environment, another source of early life adversity, in determining the risk of both cardiovascular disease and hypertension. While this has been extensively reviewed elsewhere [84,85,86] it is worth noting that the relative risk associated with birthweight and ponderal index is by far larger than any other risk factor identified for either disease to date. There is now a large body of evidence showing diabetes to be a major risk of complications and death after SARS-CoV-2 infection [87], as in previous coronavirus outbreaks [88], while the risk of SARS-CoV-2 infection appears to be similar [89]. Like the other elements discussed here, type 2 diabetes (T2D) may have its origins in early life. There are well-established, classical risk factors that contribute to T2D including obesity, age, stress, inflammation, diet, lifestyle and environment (both early and late life), however there is growing recognition for non-classical factors such as pollution, exposure to ionizing radiations and low socio-economic status (SES). The classical and non-classical factors are intimately intertwined. SES is a broad measure encompassing prior life history, and low SES also increases the risk for obesity, stress, environmental and lifestyle factors (BMI, smoking, alcohol…) as well as a pro-inflammatory phenotype [90].

The importance of T2D in determining COVID-19 severity may in part be due to treatment strategies currently used in T2D together with another severe co-morbidity, hypertension. Both are often treated with ACE (angiotensin converting enzyme) inhibitors and ARBs (angiotensin II receptor blockers). These increases ACE2 (angiotensin converting enzyme 2) expression in pancreatic islets, lungs, intestines, etc. [91]. SARS-CoV-2 exploits these ACE2 receptors to enter host cells, thus potentially increasing the risk of infection in T2D patients [92]. Increased pancreatic ACE2 activation has been reported to inflict beta cell damage complicating the prognosis [93] and further contributing to the characteristic “cytokine storm” observed in COVID-19 cases. Other T2D drugs that induce ACE2 expression include pioglitazone, liraglutide, gliflozins, and DPP4 (dipeptidyl peptidase 4) inhibitors and have also been implied to promote coronavirus predisposition [94]. This may be further accentuated by hyperglycemia-induced ACE2 glycosylation. ACE2 glycosylation is also a prerequisite for the virus to latch onto the ACE2 receptors [95]. This enhancement is reversible by strict glycemic control [95]. As such, glycemic and overall diabetic status have been proposed as predictors of COVID-19 severity and mortality [96].

Although current T2D status may play an important role in SARS-CoV-2 susceptibility and COVID-19 severity, it is part of a larger etiopathological risk complex. T2D may have its origins in the early life social environment. Low early-life SES showed a clear, strong, association with individual metabolic profiles that was not true for current SES [97]. This result has been replicated by another study that highlighted the effect of SES during adolescence on the development of T2D up to fifty years later [98]. More recently, Chandan et al. (2020) reported a retrospective population-based cohort of 80,657 adults that had been exposed to ELA and 161,314 unexposed controls. This seminal study clearly demonstrated the link between childhood maltreatment and cardiovascular disease, hypertension, and T2D. In a population where ELA rates may reach 25%, their data clearly shows that “a significant proportion of the cardiometabolic and diabetic disease burden may be attributable to maltreatment” [99].

There is now some mechanistic evidence to back up the link between ELA and T2D. Needham et al. investigated the transcriptional effects of low SES [100]. They reported that low (current) adult SES altered the expression of several genes intimately linked to inflammation that are all linked to T2D: *F8* [101], *CD1D* [102], *KLRG1* [103], *NLRP12* [104], and *TLR3* [105] and stress related gene *AVP* [106]. Furthermore, low early-life SES was also shown to affect the expression of stress related genes: *FKBP5* [107] *OXTR* [108] and *AVP* and inflammation associated genes: *CD1D* and *CCL1.* As such, SES would appear to act on inflammatory pathways that are common to low SES environments and eventually T2D, and may worsen the T2D etiopathology by targeting prominent pathophysiological factors like stress and inflammation. The mechanistic link between ELA and T2D is re-enforced by the immune disturbances reported. Patients with T2D have a larger number senescent CD8+ cytotoxic T cells and higher levels of systemic inflammation [109,110] that may explain the higher incidence of viral and bacterial infections in diabetic patients [111].

Although there is no data currently available, it is logical to assume that although T2D may predict COVID-19 severity, the origins of this link may lie in the lifelong pro-inflammatory environment induced by ELA. T2D may be the adult manifestation of the poor early life social environment which then mediates the effect between ELA and COVID-19.

## 5. The COVID-19 Immune Response, SES and Early Life Adversity

**Figure 2:**
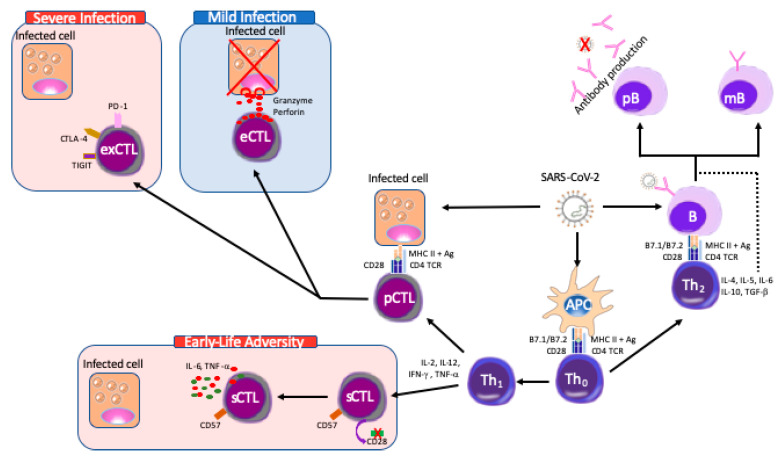
The immune reaction to coronavirus disease (COVID-19). The adaptive response to SARS-CoV-2 is a classical anti-viral response. On the right side, once recognized by antigen presenting cell (APC), Th_2_ response is activated and induced maturation of B cell. After maturation precursor B cell produces a specific antibody against SARS-cov-2 while mature B cell retain memory of SAR-COV-2 to produce antibodies in case of new infection. Once the Th1 system is activated it induces activation of precursor cytotoxic lymphocyte T (pCTL) due to expression of many cytokines (IL-12, IL2). In one hand, effector (eCTL) can release proteins as granzyme to destroy infected cell in case of mild infection. In case of severe infection, CTL become exhausted (exCTL) and express PD-1, TIGIT and CTLA-4. In patients with having experienced ELA, the increased relative number of sCTL having lost CD28 expression will produce a less efficient lysis of SARS-CoV-2 infected cells. The recognition and clearance by NK cells and the initial role if Interferons is omitted for clarity. Cell images were from http://www.clker.com with the right to re-use them.

*The immune response to COVID-19:* The SARS-CoV-2, like other viruses, is considered immunologically as an intracellular parasite. In general, the viral infectious-cycle starts with a short-lived extracellular period, followed by cell entry, with a final, longer, intracellular replicative period. In the classical anti-viral immune response, the immune system attacks all phases of the viral cycle using both antigen specific and non-specific mechanisms. The non-specific immune response, particularly effective in the early phase of infection, is mainly mediated through natural killer cells and interferons. Production and/or secretion of type-1 interferons (i.e., all the interferons proteins except IFN-γ) enhances NK cell ability to lyse infected cells as well as inhibits viral reproduction and cellular proliferation. When an adaptive immune response has been mounted, the most effective antibodies are the so-called neutralizing antibodies which block viral entry into the host cell by binding to viral surface proteins such as the envelope or capsid protein (Figure 2). When the subsequent cell-mediated immunity enters into force, it is principally CD8+ cytotoxic T lymphocytes (CTLs) that are the effector cells. CTLs recognize MHC class-I presented antigens, to lyse the presenting cell, a response that is not always beneficial as the damage done by the cytotoxic cells is occasionally greater than that of the virus itself.

As the COVID-19 pandemic has progressed, there have been several reports of the anti-SARS-Cov-2 immune response. To date, the data suggests that the response is a classical anti-viral response with activation of Type-1 interferons and CD8+ CTLs. Although Thevarajan et al., analyzed a single patient, they nicely demonstrated the kinetics of the anti-SARS-CoV-2 immune response [112]. In a manner similar to both Influenza infection and a previous SARS-CoV-2 report [113] which showed that the numbers of CD38^+^HLA-DR^+^ CD8^+^ T cells were higher in infected patients than in healthy controls, and rapidly increased from 3.57% (day 7), 5.32% (day 8) to a peak at 11.8% 9-days later. By day 20 they had decreased slightly to 7.05%. As would be expected, CD38^+^HLA-DR^+^CD8^+^ CTLs, produced significant quantities of the lytic moieties—perforin, granzyme A and granzyme B—necessary to lyse virus-infected cells (Figure 2). Their kinetic data showed that this occurred at days 7–9, preceding symptom resolution, suggesting an important role in the resolution of the SARS-CoV-2 immune response [112].

*The anti SARS-CoV-2 immune response in severe/critical patients:* COVID-19 patients are generally considered either mild, severe, or critical. There are now data on the differences in the immune response in these different categories, although the categories are not always the same, complicating comparisons between studies. When Zheng et al. investigated T-cell derived functional molecules, they highlighted lower levels of interferon-γ (IFN-γ) and TNF-α in CD4+ T cells in severely affected patients than those mildly affected, although in the latter, they were considerably higher than expected in health controls [114]. Levels of perforin and granzyme B cells were increased in CD8+TIGIT- CTLs, and the numbers of senescent HLA-DR+ TIGIT+ CD8+ cells were increased in severely affected patients than those with a mild infection. The authors proposed that their data suggests COVID-19, like many chronic viral infections, reduces CD4-Tcell functionality, skewing the immune response towards a CD8+ response, with excessive activation leading to exhaustion of the CD8+ cells, diminishing the anti-viral immune reaction. Furthermore, upon deeper examination, they found differences in PD-1, CTLA-4, and TIGIT–markers of immune exhaustion. In severely affected patients, exhausted PD1+CTLA-4+TIGIT+ cells were significantly more frequent than in patients with a milder infection. This excessive CTL exhaustion may reduce the effectiveness of the immune response to SARS-CoV-2, explaining case severity [114]. Furthermore, in an independent study, it was also reported that as disease severity increases, the numbers of naïve, effector and memory classes of CD8+ T cells diminish, while B-cell, and CD4+ T cell numbers generally increase [115,116]. Overall, we interpret these data as showing that a functional CD8 response is required to clear SARS-CoV-2 infection, and COVID-19 severity is increased as the CD8+ response becomes somehow diminished (Figure 2). Indeed, Omarjee et al. have also come to a similar conclusion, that “Severe COVID-19 can therefore mimic a state of immune senescence” [117,118]. From the start of the pandemic, the involvement of the cytokine system was clear [119]. Initially described in January 2020, levels of CXCL8 and IFNγ, were increased in all COVID-19 patients, and severe cases had significantly higher levels CXCL10, CCL2 and TNFα than milder cases [120] reproduced in a more recent study that also observed increased levels of IL6, and IL 10 in the most severe cases [121].

*Does Immunosenescence link ELA to COVID-19 outcomes?* We have outlined above the ELA-induced long term immunophenotype. Although the origins are multifactorial, it would appear, from the work of Elwenspoek [27,28] and Reid [29], that an adverse social environment in early life drive T-cells, in particular CD8+ CTLs, towards a senescent state. When the different aspects of ELA are considered separately, immunosenescence would appear to be a common aspect. Senescence and exhaustion may have similar outcomes, a reduced immune reaction, but are distinct processes [122]. Senescent cells have a significantly reduced capacity to proliferate, however, they have a strong pro-inflammatory action. In a manner reminiscent of the senescence associated secretory phenotype (SASP) initially established in fibroblasts [123] senescent CD8+ CTLs aggregate in the highly differentiated states (effector memory and TEMRA), are highly resistant to apoptosis, and produce significant quantities of pro-inflammatory cytokines such as IL6 and TNFα upon stimulation [124]. Exhausted CD8+ CTLs however, are not only unable to proliferate, but they no longer secrete cytokines after stimulation and are programed to undergo apoptosis.

The data currently available suggests that the aggregation of senescent CTLs will negatively impact the progression of COVID-19, and patients with the most senescent CTLs will have the poorest prognosis as they are less capable of mounting an effective CD8+ response, and they will have an exaggerated cytokine secretion from the senescent cells. This is further supported by the recent initiation of the SCOPE trial, “Sirolimus Treatment in Hospitalized Patients With COVID-19 Pneumonia” (NCT04341675). In this trial, the investigators propose administering rapamycin to down-regulate the IL-6 pathway through the mTOR pathway to not only reduce IL-β levels, but reduce the number of senescent T-cells as well [117]. This also raises the question about what happens to COVID-19 when ELA-induced senescence and COVID-induced exhaustion converge. It would seem logical to hypothesize that this would represent the worst-case scenario, and would produce the least-effective cytotoxic T cell response.

*The Conserved Transcriptional Response to Adversity (CTRA):* Studies have demonstrated that early life social adversity can act mechanistically through modifications of gene expression patterns. Gene expression implicated in the activation of T-lymphocyte and inflammation was enhanced while gene expression implicated in innate antiviral responses induced by type I IFN and innate antimicrobial responses of pathogen-specific was reduced [125]. These patterns of altered gene expression remain lifelong [125]. The pattern has been termed the conserved transcriptional response to adversity (CTRA), and has been noticed in many correlational studies regarding humans encountering with adverse life circumstances. [126,127,128,129,130,131,132,133]. CTRA dynamics are most strongly induced by social conditions in early life, at the first step of the development of postnatal immune system [125]. To the extent that transcriptome remodeling induced environmentally continue to affect immune responses of implicated pathogen, many, many years later in life (e.g., inhibiting immune responses to viral infections [134], or amplifying allergic inflammation [133,135]).

*Essential co-variates:* ELA is, however, associated with a range of negative health behaviors (reviewed in [136]) including an increased risk of smoking as well as increased smoking levels, levels of alcohol consumption, and poor diet leading to either malnourishment or obesity. The psychobiological and neurodevelopmental mechanisms linking ELA and risky health behaviors are starting to be dissected [137]. However, in the context of the COVID-19 pandemic, it would appear from the numerous studies that are becoming available that smoking increases the risk not only of hospitalization with COVID-19, but with ICU admission and death (odds ratio from 2.0 to 16 [138,139]) and was confirmed in recent meta analyses of the available studies [140,141,142,143]. On the other hand, there is little evidence available on the role of prior alcohol intake on the course of SARS-CoV-2 infection, however, considerable public health efforts are being made to combat alcohol abuse during the confinement period, and a prior history of ELA exposure may increase the risk of excessive alcohol consumption during this period.

Biological sex is one of the strongest drivers of the heterogeneity in COVID-19 disease severity. There is a clearly more favorable outcome for women across all age categories. The data available so far suggests that sexual dimorphism in the immune system may play a role in determining disease outcome. Sex impacts not only the development of T_reg_ cells, but the distribution of lymphocyte subsets and the overall T-lymphocyte response to challenge [144]. Many immunologically important genes are found on the X-chromosome including CD40L and CXCR3. Incomplete X-inactivation or epigenetic modifications will induce sex-specific effects on T-cells [145,146]. There is also evidence that there is a stronger lymphopenia in males than females in severe COVID-19 disease [147,148]

There is also growing evidence for the role of vitamins D and K in the outcome of COVID -19 disease. Beyond its classical role in bone metabolism [149], vitamin D plays a role in the functioning of the immune system and in the regulation of inflammatory cytokines [150] and CRP [149] which reduces the risk of infection and cardiovascular disease [149]. Indeed, immune cells like T-cells, B-cells or antigen presenting cells can directly interact with vitamin D receptors. In this way, increased vitamin D levels enhance the innate system and suppress the adaptive immune system, which demonstrates its role in immune regulation [151]. Vitamin D deficiency is also linked to comorbidities such as diabetes [152] and upper respiratory disease susceptibility, including common viral infections, allergies and airway inflammatory conditions (REF6). The logical assumption is that a possible explanation on the susceptibility of the elderly population is the fact that they naturally produce less vitamin D while they are exposed to less sunlight as many stay indoors. Considering also that the pandemic first made its global appearance during winter season increases the possibility for this correlative association [152]. Panfili et al. highlighted the potential that vitamin D supplementation has shown to be a successful cost-effective therapeutic for acute respiratory tract infections (ARTIs) in low socio-economic characterized countries [153]. In addition, studies have shown that vitamin D can help to reduce the risk of an activated renin-angiotensin system in the lung [154] in cases of severe COVID-19 disease in patients with hypertension and high expression of ACE2 receptors [155]. On the other hand, patients with comorbidities such as diabetes present a lack in vitamin K which is involved in blood coagulation or bone calcification mechanisms. In case of COVID-19 patients, insufficient levels of vitamin K could be associated with a risk of complications due to elastic fiber pathologies such as idiopathic pulmonary fibrosis (IPF) [156]. Coagulation has been reported as a common comorbidity linked to COVID-19 severity and mortality.

## 6. COVID-19 as a Natural Experiment

Given the obvious ethical objections to experimental studies manipulating the early life environment, there is a long history of using natural experiments. There are two classical natural experiments looking at the early life social environment, Project Ice Storm in Canada, and the Dutch Hunger Winter. When we look at these natural experiments in the light of the three-hit model, these examined the role of the second hit, the early life environment.

Project Ice storm is based on the 1998 Quebec ice storm and examines the impact of prenatal stress on adult outcomes. This particularly harsh meteorological event affected, residents of a well delineated area covering Nova Scotia, New Brunswick, Southern Quebec and eastern Ontario. These populations had to deal with a situation where they were deprived of electricity for weeks, and in certain cases months, as well as the shutdown of all activities in major cities (Montreal, Ottawa) as well as military deployment and several deaths. Project Ice Storm went on to examine the effects over the following 20 years on the children and now young adults that were exposed to the storm in utero [157,158]. They concluded that prenatal glucocorticoid exposure impacted a variety of outcomes in the next generation throughout childhood and persisting into adulthood, dysregulating metabolic pathways and the HPA axis [157,159] This was mediated through epigenetic (DNA methylation) encoding of the storm’s effect [158]. Project Ice Storm demonstrated that an environmental stressor can have long-term effects and inducing numerous outcomes although there were additional mechanisms linked to socioeconomic factors that are still to be identified.

The Dutch Hunger Winter was the consequence of a food embargo placed on the Dutch population by the Germans at the end of world war II [160]. Here, the importance of timing of the adversity in the programing of adult disease was established [161]. Working on same-sex sibling pairs of which only one was exposed to famine they demonstrated that in utero exposure induced an adverse metabolic [162] or mental phenotype [163], depending on the time of exposure and fetal sex, and that this was mediated by DNA methylation [164].

As Project Ice Storm disaster and the Dutch hunger winter, the current COVID-19 pandemic must be considered as a relevant natural experiment to reveal the effects of socioeconomic factors on health and disease. In the context of the three-hit model, here we have an exquisite and unique opportunity to investigate the third hit. As outlined above, the early life period acts through underlying mechanisms such as DNA methylation and programing of the immune system to influence disease progression and severity later in life. These prior studies have provided unexpected mechanistic insight into the immunological consequences of early life stress exposure. Drawing parallels with COVID, if we can collect the correct data, we can start to unpick the role of the whole life trajectory and how this contributes to disease risk through a pro-inflammatory immune bias.

COVID-19 may also be a form of early life adversity. It is yet to be discovered whether SARS-Cov-2 could have any immune programing capacity after an early life infection and what consequences could appear years later. Its strong association and impact on the early life microbiome is unknown. Pregnant women who tested positive for SARS-CoV-2 infection showed evidence of placental injury which impeded blood flow to the fetus [165]. Placental development is the first step in embryogenesis and may determine the quality of the intra-uterine environment [165,166]. Individuals who were exposed (intra-uterine) to the Spanish flu of 1918 have been reported to face lifelong low SES and cardiovascular diseases [167] which may be indicative of a bidirectional risk that has crossed over from the placenta jeopardizing their lifelong health profile. It is quite possible that the COVID-19 positive mothers pass on a similar risk to subsequent generations, serving as an ELA event, which ultimately makes them highly susceptible. Thus, these cases need strict follow up studies to validate this hypothesis.

## 7. Data that Should Be Collected

In light of the data presented here, it is clear that there are many types of data that should be collected in addition to the studies that are currently ongoing addressing the epidemiology and biology of COVID-19. As recently highlighted, it is essential to collect as much socioeconomic data as possible during the ongoing pandemic [16]. Data collection should be expanded to include retrospective data on life-trajectories and both exposure to adverse life events and how importantly they were perceived. There are well-recognized difficulties in retrospectively assessing adversity or the overall life-course, however, there are tools available that can measure the prior traumatic experiences. Recent adult trauma can be addressed by a brief questionnaire that covers the perceived importance (salience) of a range of stressful life events including “separation, relationship and money worries, accidents, illness and death, job loss, and violence” [168] that any future study participants may have experienced. To address traumatic experiences earlier in life, there are also validated questionnaires such as the Childhood Trauma Questionnaire CTQ or the Early Trauma Index that are available [169]. However, as with any retrospective study there is a risk of recall bias, although the validated questionnaires have questions within them to ensure internal consistency. Furthermore, in the context of a fast-moving pandemic, the ability to transpose such questionnaires to an online system is known to improve the accuracy of responses as the anonymity of the online process has been shown to reduce both social desirability and central coherence biases, although there is a potential risk of questions being mis-interpreted by participants [170]. All such tools are limited by what was thought of as being traumatic when they were developed, however, they remain the standard tool for assessing traumatic events during childhood as well as a poor social and familial environment [169]. The use of such questionnaires has already proven useful. Adverse social conditions, as measured by the CTQ have been shown to become embedded as functional changes in the immune system that are visible lifelong. Studies have shown adversity measured by the CTQ over a period of as little as 4 months changes the immune response up to 24 years later, the longest time-point investigated so far [27,28,34,125]. Tools such as the CTQ should play a role in studies addressing the overall disease severity if participants go on to develop COVID-19 rather than whether ELA plays a role in the overall prevalence of infection. Furthermore, health related behaviors such as smoking and alcohol consumption which are known to be elevated after ELA and may also play a role in the clinical evolution or susceptibility to SARS-CoV-2 infection must be recorded. All data should be analyzed with a sex-informed approach, taking differences in the immune system into account.

The collection of life-event meta-data must be complemented by the collection of the correct biological samples. We have highlighted the role of the immune system, the microbiome and pollution levels. It would seem logical to obtain stool and blood samples, and the markers to be investigated such as TIGIT, PD-1, CD28 and CD57 are now becoming clear. Furthermore, such biosampling would allow the analysis of vitamin levels, as they may be a key link in the pathophysiological chain. It would also appear to be appropriate to rapidly collect measures of pollutants, determine how indoor and outdoor pollution levels have changes, how, with the strict confinement measures imposed, nutrition has changes. All of these will play into the susceptibility and immune response.

The data reviewed here highlights the role that the social environment will play in determining morbidity and mortality during the COVID-19 pandemic. In the future, such socioeconomic and lifestyle data must be considered as essential clinical data that is then analyzed concurrently with biological material to tease out the effects of the environment in health and disease.

## 8. Conclusions

The developmental origins of health and disease is firmly established for many non-communicable diseases. The current COVID-19 pandemic has shown that there are many health disparities, and the available (preliminary) data suggests that there is a strong socioeconomic impact on morbidity, and potentially mortality. Although there are no data so-far available to link the early life period to the morbidity and mortality of an infectious disease, an adverse early life environment would appear to impact the immune system and make it less efficient in fighting subsequent viral infections. Early-life researchers have a long history of taking advantage of natural experiments, teasing out the long-term consequences of ELA to produce a measurable phenotype many years, or even generations, later. The current pandemic can turn this paradigm on its head. Many discrepancies and inequalities in COVID-19 morbidity and mortality have been reported, and if the correct data is collected it may be possible to separate the early life elements that have made people particularly vulnerable to COVID-19 many years later. This will, naturally, then help us identify those that are most at risk from developing the severest forms of COVID-19. In order to do this, we need to recognize socioeconomic and early-life factors as genuine medically and clinically relevant data that urgently need to be collected. Finally, many biological samples have been collected in the ongoing studies. The mechanisms linking the early life environment with a defined later-life phenotype are starting to be elucidated, and perhaps hold the key to understanding inequalities and differences in the severity of COVID-19.
